# Prevalence of Dental Caries and Periodontal Disease of High School Students Aged 15 to 18 Years in Taiwan

**DOI:** 10.3390/ijerph18199967

**Published:** 2021-09-22

**Authors:** Po-Sen Chang, Chun-Jui Huang, Chia-Lan Hsiang, Hongmin Lai, Aileen I. Tsai

**Affiliations:** 1 Department of Dentistry, New Taipei Municipal Tucheng Hospital, New Taipei City 236, Taiwan; bosen@cgmh.org.tw; 2 Department of Pediatric Dentistry, Chang Gung Memorial Hospital, New Taipei City 333, Taiwan; 3 Division of Endocrinology and Metabolism, Department of Medicine, Taipei Veterans General Hospital, Taipei 112, Taiwan; cjhuang3@vghtpe.gov.tw; 4 Faculty of Medicine, School of Medicine, National Yang-Ming University, Taipei 112, Taiwan; 5 Eledent Kid’s Dental Clinic, Taipei 114, Taiwan; hsiangcl@gmail.com; 6 Dentway International Dental Hospital, Taipei 114, Taiwan; 7 Dental Department, Taipei Medical University Shuang-Ho Hospital, New Taipei City 235, Taiwan

**Keywords:** dental caries, periodontal disease, national dental survey, community periodontal index, loss of attachment

## Abstract

The purpose of this study was to assess the prevalence and distribution of dental caries and periodontal disease in Taiwanese high school students aged 15–18. A total of 1069 Taiwanese students participated in a cross-sectional nationwide dental survey. By using a stratified method based on the National Health Insurance administration regions, 24 high schools were randomly sampled in different areas of Taiwan. The participants were examined with dental mirrors and community periodontal index (CPI) probes without using radiographs, to measure dental caries and periodontal status. Demographic information and other relevant risk indicators for the two diseases were gathered via a self-report questionnaire. In this study population, the weighted mean decayed, missing and filled teeth (DMFT) scores for ages 15 to 18 were 5.2, 6.1, 6.1, and 5.7. The weighted mean decayed, missing and filled surfaces (DMFS) scores were 9.0, 12.0, 13.1, and 11.2 at ages 15, 16, 17, and 18, respectively. Additionally, 88.2% of the subjects had periodontal disease, with calculus as the most prevalent type. Moreover, 5.2% of these students showed loss of attachment. There was no significant association between dental caries and periodontal disease. Dental caries and periodontal disease were prevalent among Taiwanese high school students in this national dental survey.

## 1. Introduction

Over the past thirty years, the economic situation in Taiwan has positively progressed considerably, and the health status of the people has also greatly improved [1]. However, the oral health situation is one exception to these tandem advances.

Dental caries and periodontitis are a dental public health issue affecting the whole world [2,3,4,5] and represent the major cause of tooth loss [6,7]. Both dental caries and periodontal disease status are two major items included in dental health surveys. Dental caries involves the complex interplay between the tooth surface, bacteria, and a carbohydrate source [8]. Periodontitis is the inflammation of periodontal tissues, resulting in the formation of pockets, loss of connective tissue attachment, and destruction of alveolar bone [9].

Epidemiological studies have demonstrated that the prevalence of dental caries in elementary and junior high school students in Taiwan is approximately 94% [10]. However, only a few studies have documented the condition of dental caries in high school-age populations in Taiwan.

Adolescents are susceptible to periodontal diseases [11,12]. A global epidemiological review on the periodontal status of adolescents showed that the prevalence of periodontitis ranged from 1–20% either by country or by race [13]. The 15- to 19-year-old group represented one of the key age groups for epidemiological surveys, given that these teenagers begin to be responsible for their health and lifestyles at that age [14]. The most prevalent periodontal condition in this age group is calculus, followed by gingival bleeding [14,15]. There are many available indices to survey periodontal diseases. The Community Periodontal Index of Treatment Needs (CPITN) was advocated by the World Health Organization (WHO) in 1982, because it is simple, quick, and inexpensive [16]. It has been widely used in many countries [12,14,15] including Taiwan [17,18]. In 1988, a Taiwanese survey based on CPITN reported that 86.6% of 15- to 19-year-old students had periodontal disease, with calculus being the most prevalent problem [17]. Another Taiwanese CPITN study in 1990 demonstrated that 80.7% of 13- to 20-year-old students had periodontal disease [18].

Our 2008 national periodontal survey showed 21.9% of adults aged 18 to 24 years already had periodontal pockets [19]. To tackle this problem and develop an effective prevention program, we need complete data on the dental conditions of our younger generations.

The scarcity of dental data regarding adolescents between aged 15 to 18 makes it difficult to obtain firm conclusions on the prevalence of dental caries in this population. Furthermore, there are even fewer data about the periodontal condition of 15- to 18-year-old adolescents living in Taiwan. Thus, this national dental survey of adolescents was conducted from late 1999 to early 2001 to establish new data for designing effective preventions and treatments in dentistry as it applied to the adolescent population in Taiwan.

Therefore, we think these data are still relevant today and resume the analysis of the national survey carried out between 1999 and 2001. The purposes of this survey were to assess the prevalence and distribution of dental caries and periodontal disease in students aged 15 to 18 years.

## 2. Materials and Methods

This cross-sectional dental study was part of the National Dental Survey of a population aged 6 to 18 years that was sponsored by the Department of Health of Taiwan. The survey was conducted from late 1999 to early 2001. This investigation was approved by the Institutional Review Board of Chang Gung Memorial Hospital (IRB number: 201600095B0C502). The target population of the periodontal survey was 1.2 million students aged 15- to 18-year-old from senior high schools or equivalent school settings. There are 309 cities, townships and villages in Taiwan. These areas were divided into six divisions of national health insurance administration as follows: (1) Taipei division, (2) Northern division, (3) Central division, (4) Southern division, (5) Kaoping division, and (6) Eastern division.

The sampling design involved a four-strata cluster sample selected with probabilities that were proportional to size. The four strata included (1) primary sampling units, which were the six insurance divisions in Taiwan; (2) townships, such as cities, towns, and villages, in each zone; (3) schools in each township; and (4) eligible students in each school. The townships were randomly selected, after which schools were selected. In each selected school, the year and gender were stratified, and the students were randomly selected.

A total of 1069 students (463 males and 606 females) from 24 high schools (or equivalent settings) were selected for dental examinations. Each participant answered a questionnaire regarding demographics, family background, lifestyle, oral hygiene habits, dental awareness, and previous dental care.

The WHO has added loss of attachment (LA) to the CPITN and created the new version, known as the community periodontal index (CPI) and LA [20]. Therefore, we used the CPI and LA as the measurement tools in this study. The following WHO criteria are briefly summarized. The CPI scores were defined as follows: 0 for healthy periodontium, 1 for gingival bleeding, 2 for calculus, 3 for shallow pockets of 4–5 mm, and 4 for deep pockets of 6 mm or deeper [16]. The LA was assessed using the cementoenamel junction (CEJ) as the reference point for measuring the extent of attachment loss and assigns scores as follows: 0 for 0–3 mm, 1 for 4–5 mm, 2 for 6–8 mm, 3 for 9–11 mm, and 4 for 12 mm [20]. X was used as the excluded sextant because it had fewer than two functional teeth; in addition, nine for the sextant were not recorded. The dentition was divided into six sextants according to the distal surfaces of the canines [20]. For subjects aged under 20 years, the following six index teeth were examined: the first molar in each posterior sextant, tooth 11 (FDI Notation) for the maxillary anterior sextant (second sextant), and tooth 31 for the mandibular anterior sextant (fifth sextant). We examined the following six sites on each tooth: mesiobuccal, mid-buccal, distobuccal, mesiolingual, mid-lingual, and distolingual sites. The highest score among the six sites was representative of the sextant. Subsequently, the highest sextant score was representative of the subject. The percentage of subjects under each CPI and LA score indicated the prevalence of each score (subject level). Moreover, the mean number of sextant CPI and LA scores per person represented the severity of periodontal disease (sextant level). Therefore, there were four primary outcomes: prevalence of CPI and LA and severity of CPI and LA.

One or two examiners (depending on the number of students) performed the CPI and LA surveys in each school. Examinations were performed in a field survey setting. Additionally, the dental caries diagnostic criteria were based on the WHO guidelines [20]. During the process, the student would sit on a chair, and the examination was performed with dental mirrors, CPI probes, and under natural light and/or a portable light. Five dentists performed the examination. Additionally, they received CPI and LA training and calibration. The most senior periodontist (HL) served as the bench-mark for the calibration. The weighted kappa values for interexaminer agreement ranged from 0.71 to 0.74 [21].

Because these variables were all categorical and did not fall under a normal distribution, Wilcoxon rank-sum tests were used to evaluate the periodontal status among age groups, as well as between genders. The odds ratios for periodontal status were estimated for the various risk indicators such as schools, parents’ highest educational levels, family incomes, mean numbers of annual dental visits, and frequencies of brushing and flossing. Univariate logistic regression analyses were used to estimate the odds ratios. Subsequently, only those factors exhibiting significant effects were entered into the multivariate logistic regression analyses. Correlations were used to explore the association between dental caries and periodontal disease. The α level was set at 0.05. The statistics program that was used for the analyses was SAS statistical software, version 9.1 (SAS institute, Inc., Cary, NC, USA).

## 3. Results

A total of 1069 students were examined. Their CPI and LA scores are shown in Table 1 and Table 2. There was no statistically significant difference among age groups or between the genders. Overall, only 11.8% of the subjects had healthy periodontal scores, i.e., 88.2% of the subjects had periodontal disease (to some extent). Moreover, calculus was the most prevalent problem, being observed in 79.2% of the subjects. The percentage of students with pockets of 4–5 mm was 5.2% in all of the age groups, whereas that the students with pockets of 6 mm or deeper was 0.1%. The CPI sextant level (severity) demonstrated that the calculus was also the most prevalent sign, given that half of the six sextants per person had calculus. Most of the subjects (94.8%) did not have LA, 5.0% of the subjects already had LA of 4–5 mm, and 0.2% of the subjects had loss of attachment greater than 6 mm. The LA sextant level demonstrated the same trend.

The distribution of the severity of periodontal conditions demonstrated that anterior sextants were better than posterior sextants. For the CPI score 0 (healthy), 65% was observed in the maxillary anterior sextant, and 46% was observed in the mandibular anterior sextant, whereas 22–38% was observed in the posterior sextants. Only 0.2–0.5% of the anterior sextants had LA, whereas 2–2.3% of the posterior sextants showed loss of attachment. Additionally, the upper arches suffered more than the lower arches regarding the severity of LA.

For the CPI status, only one question (“Do you always visit the same dentist”?) had a significant odds ratio. Additionally, those individuals who did not visit the same dentist had 1.1 times higher CPI scores than those individuals who did. Oher factors, including demographics, family background, lifestyle, oral hygiene habits, and dental awareness, did not have statistically significant odds ratios. For the LA status, only two factors obtained significance level. One factor was attending school, and the other was the frequency of dental visits. The students at vocational schools had 1.8 times higher LA scores than those students in regular high schools. Those students who frequently visited dentists due to dental problems had a 2.86 times higher (95% confidence interval: 1.01, 8.11) chance of loss of attachment than those students who did not see dentists at all. None of the CPI and LA risk indicators showed a significant trend for prevalence or severity in the multivariate regression analyses.

Table 3 shows that the mean decayed, missing, and filled teeth (DMFT) scores for ages 15 to 18 were 5.2, 6.1, 6.1, and 5.7. The corresponding decayed, missing, and filled surfaces (DMFS) scores were 9.0, 12.0, 13.1, and 11.2 for ages 15 to 18. The prevalence of dental caries seemed to increase with age, reaching a peak at 16 years of age.

There was no significant association between dental caries and periodontal disease at the subject level on either the CPI or LA. Although some posterior and anterior sextants had significant associations, the r values were very small, ranging from 0.02–0.04 for CPI and from −0.04–0.01 for LA.

## 4. Discussion

Fifteen- to 18-year-old high school students were included in this study because all their permanent teeth had completely erupted, and they had the ability to care for themselves. We chose the CPI and LA as the methods for the epidemiological survey of periodontal disease because of their specific advantages, including ease of calculation, simple equipment, quick examination time, international uniformity, and widespread use.

There have been only a few national dental surveys performed in Taiwan. The most distinctive feature of this national survey involved a change in the examination tool. The previous sharp dental explorer was replaced with the CPI probe recommended by the WHO. There is a difference in the diagnostic sensation of the sharp explorer and the CPI probe, as the CPI probe tip has a ball that is 0.5 mm in diameter [20]. Thus, the detection of dental caries would be more conservative than with the use of a sharp explorer. Therefore, the comparison of the prevalence of dental caries between the previous study and this study should be interpreted with caution. In addition, the use of a sharp dental explorer has been criticized for a long period of time. Because infectious cariogenic bacteria can be transmitted from one area to another, the area that can remineralize will also be destroyed [20,22]. Additionally, blockage of the remineralization of incipient caries due to an epidemiological survey should be avoided.

Our study, similar to most other studies [14], found that 88.2% of Taiwanese teenagers aged 15- to 18-year-old already had some extent of periodontal disease, with calculus as the most common problem. Pocketing and LA occurred in 5.3% and 5.2% of the subjects, respectively. The information on LA in this age group was scarce. These findings were very similar to two Taiwanese CPITN studies, which reported prevalence between 87% and 81%, and calculus as the most prevalent problem [16,17]. However, data from the Periodontal Profile of the Main Page of the WHO exhibit a very wide range of CPI prevalence and severity in different countries and areas [23]. Moreover, the prevalence of disease ranged from 18–100%, and the severity ranged from 0.3–5.9 sextants [23]. Additionally, our teenagers had a slightly higher prevalence in the WHO data. However, caution should be taken when interpreting the latter, which included wide ranges of periodontal disease regarding subject and sextant levels, because the study populations, examiners, and settings could have been very different. Nevertheless, almost all the studies demonstrated that risk factors of periodontal disease can present in teenagers.

In addition, age, gender, oral hygiene habits, and socioeconomic status were also associated with periodontal disease [24]. A recent Taiwanese study also showed that these factors are risk indicators for periodontal pockets in adults aged 18 years and older [19]. However, other factors, including demographics, family background, lifestyle, oral hygiene habits, and dental awareness, were not statistically significant in our study.

A positive association between dental caries and periodontal disease among adults 18 years and older has been previously reported [25,26,27,28,29]. For example, Hyman et al. found a weak positive correlation between LA and interproximal caries in adults aged 20- to 49-year-old [25]. Additionally, Frentzen et al. showed that CPITN scores of up to 3 (subject level) and DMFS (surface level) correlated in 18- to 30-year-old adults [26]. Strauss et al. reported that adults aged 35 to 44 with three or more teeth with untreated caries are more likely to get periodontitis [27]. However, this association between dental caries and all signs of periodontal disease or pockets was vague in our study.

Within the limits of this study, we used the DMFT, DMFS, CPI and LA as the measurement tools. However, the International Caries Detection and Assessment System (ICDAS) is universally accepted nowadays. Plaque is an important factor in both caries and periodontal disease. Further investigations are warranted by using the ICDAS and plaque index as measurement tools at the community level to examine the association between plaque and these two oral diseases.

## 5. Conclusions

In conclusion, the findings of this national dental survey demonstrated periodontal disease and dental caries were prevalent among high school students in Taiwan. This implies that promoting comprehensive care through oral health policies and clinical practices in order to prevent periodontal disease must be initiated in early teenage years.

## Figures and Tables

**Table 1:** The prevalence and severity of CPI ^†^ among Taiwanese students aged 15–18 years.

Age (Year)	No. of Subjects	Prevalence (%) *					Severity (# of Sextant) *					
		0	1	2	3	4	0	1	2	3	4	X, 9
15	249	9.7	3.9	82.0	4.4	0	2.4	0.5	3.0	0.1	0	0.2
16	386	11.2	2.9	81.6	4.0	0.3	2.3	0.5	3.0	0.1	0	0.2
17	334	12.4	3.8	76.6	7.2	0	2.3	0.5	2.9	0.1	0	0.3
18	100	17.4	5.4	71.8	5.4	0	2.4	0.5	2.9	0.2	0	0.1
Total	1069	11.8	3.7	79.2	5.2	0.1	2.3	0.5	3.0	0.1	0	0.2

^†^ CPI scores: 0, healthy periodontium; 1, gingival bleeding; 2, calculus; 3, 4–5 mm pockets; 4, 6 mm or deeper pockets; X, excluded sextant (<2 functional teeth in each sextant); 9, sextant not recorded. * No statistically significant difference, according to age and gender (*p* > 0.05). CPI: community periodontal index.

**Table 2:** The prevalence and severity of LA ^†^ among Taiwanese students aged 15–18 years.

Age (Year)	No. of Subjects	Prevalence (%) *					Severity (# of Sextant) *					
		0	1	2	3	4	0	1	2	3	4	X, 9
15	249	95.2	4.4	0.4	0	0	5.8	0.1	0	0	0	0.2
16	386	94.9	4.9	0.3	0	0	5.8	0.1	0	0	0	0.2
17	334	94.9	5.1	0	0	0	5.7	0.1	0	0	0	0.2
18	100	93.5	6.5	0	0	0	5.7	0.2	0	0	0	0.1
Total	1069	94.8	5.0	0.2	0	0	5.7	0.1	0	0	0	0.2

^†^ LA scores: 0, 0–3 mm; 1, 4–5 mm; 2, 6–8 mm; 3, 9–11 mm; 4, ≥12 mm; X, excluded sextant (<2 functional teeth in each sextant); 9, not recorded. * No statistically significant difference, according to age and gender (*p* > 0.05). LA: loss of attachment.

**Table 3:** Dental caries prevalence, mean DMFT, and mean DMFS scores among Taiwanese students aged 15–18 years.

Age (Year)	% of Subjects Affected ± SD	D	M	F	DMFT ± SD	D	M	F	DMFS ± SD
15	87.9 ± 0.3	2.0	0.1	3.0	5.2 ± 3.8	3.5	0.7	4.7	9.0 ± 8.6
16	90.5 ± 0.3	2.1	0.3	3.7	6.1 ± 4.2	4.2	1.3	6.6	12.0 ± 12.0
17	89.9 ± 0.3	2.3	0.4	3.4	6.1 ± 4.4	4.9	1.9	6.3	13.1 ± 14.2
18	88.0 ± 0.3	2.0	0.2	3.5	5.7 ± 4.5	4.4	1.0	5.9	11.2 ± 12.2

DMFT: decayed, missing, and filled teeth; DMFS: decayed, missing, and filled surfaces; SD: standard deviation.

## Data Availability

The data used to support the findings of this study are available from the corresponding authors upon request.
